# Chromosome-level genome assembly of chub mackerel (*Scomber japonicus*) from the Indo-Pacific Ocean

**DOI:** 10.1038/s41597-023-02782-z

**Published:** 2023-12-08

**Authors:** Young Ho Lee, Linelle Abueg, Jin-Koo Kim, Young Wook Kim, Olivier Fedrigo, Jennifer Balacco, Giulio Formenti, Kerstin Howe, Alan Tracey, Jonathan Wood, Françoise Thibaud-Nissen, Bo Hye Nam, Eun Soo No, Hye Ran Kim, Chul Lee, Erich D. Jarvis, Heebal Kim

**Affiliations:** 1 Interdisciplinary Program in Bioinformatics, Seoul National University, Seoul, Republic of Korea; 2 Vertebrate Genome Laboratory, The Rockefeller University, New York, New York USA; 3 Department of Marine Biology, Pukyong National University, Busan, 48513 Republic of Korea; 4 Tree of Life, Wellcome Sanger Institute, Cambridge, CB10 1SA UK; 5 National Center for Biotechnology Information, National Library of Medicine, National Institutes of Health, Bethesda, MD USA; 6 Biotechnology Research Division, National Institute of Fisheries Science, Haean-ro 216, Gijang-eup, Gijang-gun, Busan, 46083 Korea; 7 Plant Systems Engineering Research Center, Korea Research Institute of Bioscience and Biotechnology, Daejeon, Korea; 8 Laboratory of Neurogenetics of Language, The Rockefeller University, New York City, NY 10065 USA; 9 Howard Hughes Medical Institute, Chevy Chase, Maryland USA; 10 eGnome inc., C-1008, H Businesspark, 26, Beobwon-ro 9-gil, Songpa-gu, Seoul, Republic of Korea; 11 Department of Agricultural Biotechnology and Research Institute for Agriculture and Life Sciences, Seoul National University, Seoul, Republic of Korea

**Keywords:** Genome informatics, Ichthyology

## Abstract

Chub mackerels (*Scomber japonicus*) are a migratory marine fish widely distributed in the Indo-Pacific Ocean. They are globally consumed for their high Omega-3 content, but their population is declining due to global warming. Here, we generated the first chromosome-level genome assembly of chub mackerel (fScoJap1) using the Vertebrate Genomes Project assembly pipeline with PacBio HiFi genomic sequencing and Arima Hi-C chromosome contact data. The final assembly is 828.68 Mb with 24 chromosomes, nearly all containing telomeric repeats at their ends. We annotated 31,656 genes and discovered that approximately 2.19% of the genome contained DNA transposon elements repressed within duplicated genes. Analyzing 5-methylcytosine (5mC) modifications using HiFi reads, we observed open/close chromatin patterns at gene promoters, including the *FADS2* gene involved in Omega-3 production. This chromosome-level reference genome provides unprecedented opportunities for advancing our knowledge of chub mackerels in biology, industry, and conservation.

## Background & Summary

**Fig. 1:**
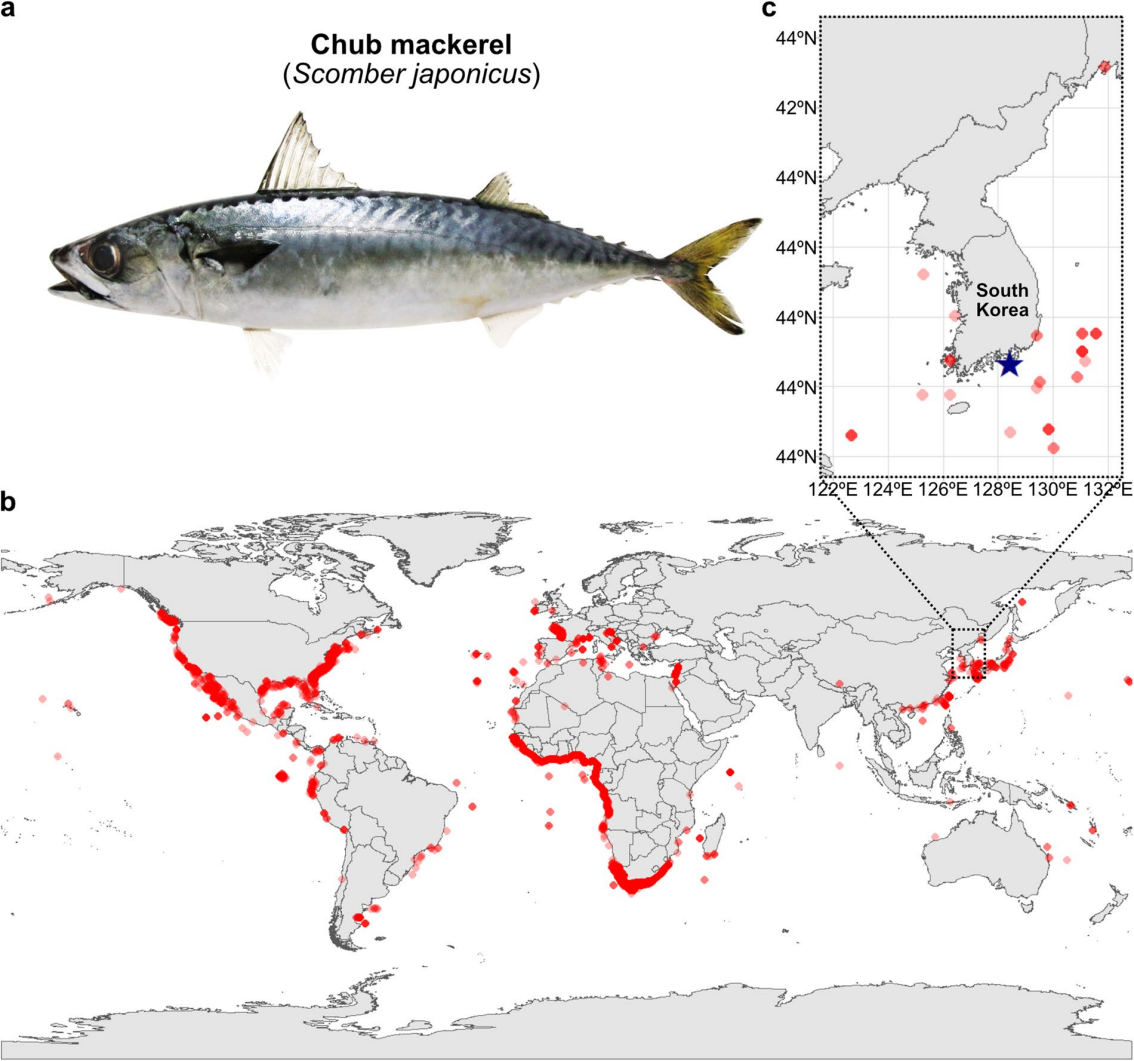
Morphological features, worldwide occurrences and sampling location of chub mackerel. (**a**) Morphology of chub mackerel provided from the Marine Fish Resource Bank of South Korea (MFRBK). (**b**) Locations of worldwide occurrences of chub mackerel. (**c**) Local map of the sampling location of the chub mackerel individual of fScoJap1 assembly marked as a blue star mark in South Korea (34°46′15.8″ N, 128°23′54.0″ E). Each red dot on the map represents an occurrence location. Some dots were shaded (30% transparency) to display overlapping dots.

Mackerels are a group of migratory, schooling, marine, coastal-pelagic fish in the family *Scombridae*^1,2^. Pacific chub mackerels (e.g. *Scomber japonicus* Houttuyun, 1782) are the primary and most widespread species of the mackerel group^3^, composing 43% of *Scombridae* landings^4^. They are classified as a distinct species from Atlantic chub mackerel (*Scomber colias*) based on differences in morphology and molecular data^5^. Chub mackerels have an elongated body^2,6^, which is dorsally pale green with faint steel blue wavy lines and laterally silvery yellow with round blotches that develop over time^7,8^ (Fig. 1a). They are characterized by two separated dorsal fins, a pectoral fin on each side, an anal fin and a caudal fin^2^. Ecologically, they inhabit temperate to subtropical waters of Pacific, Atlantic and Indian Oceans, displaying antitropical distributions^9^ (Fig. 1b). They are prey for larger pelagic fish and marine mammals^10^, playing a crucial role in the marine food chain. Commercially, this marine fish is captured and consumed worldwide^11^ and serves as significant sources of omega-3 fatty acids, which are in high demand and predominantly derived from fish oil^4^. Additionally, their population is dispersed across discrete and disjunct geographical areas^9^, making them suitable for comparative genetic studies. Despite their ecological and commercial value, the population size of chub mackerel has recently declined^11^ due to climate change affecting optimal habitat conditions and temperature-dependent hatching rates^12^, placing the genetic resources of chub mackerel at stake.

**Fig. 2:**
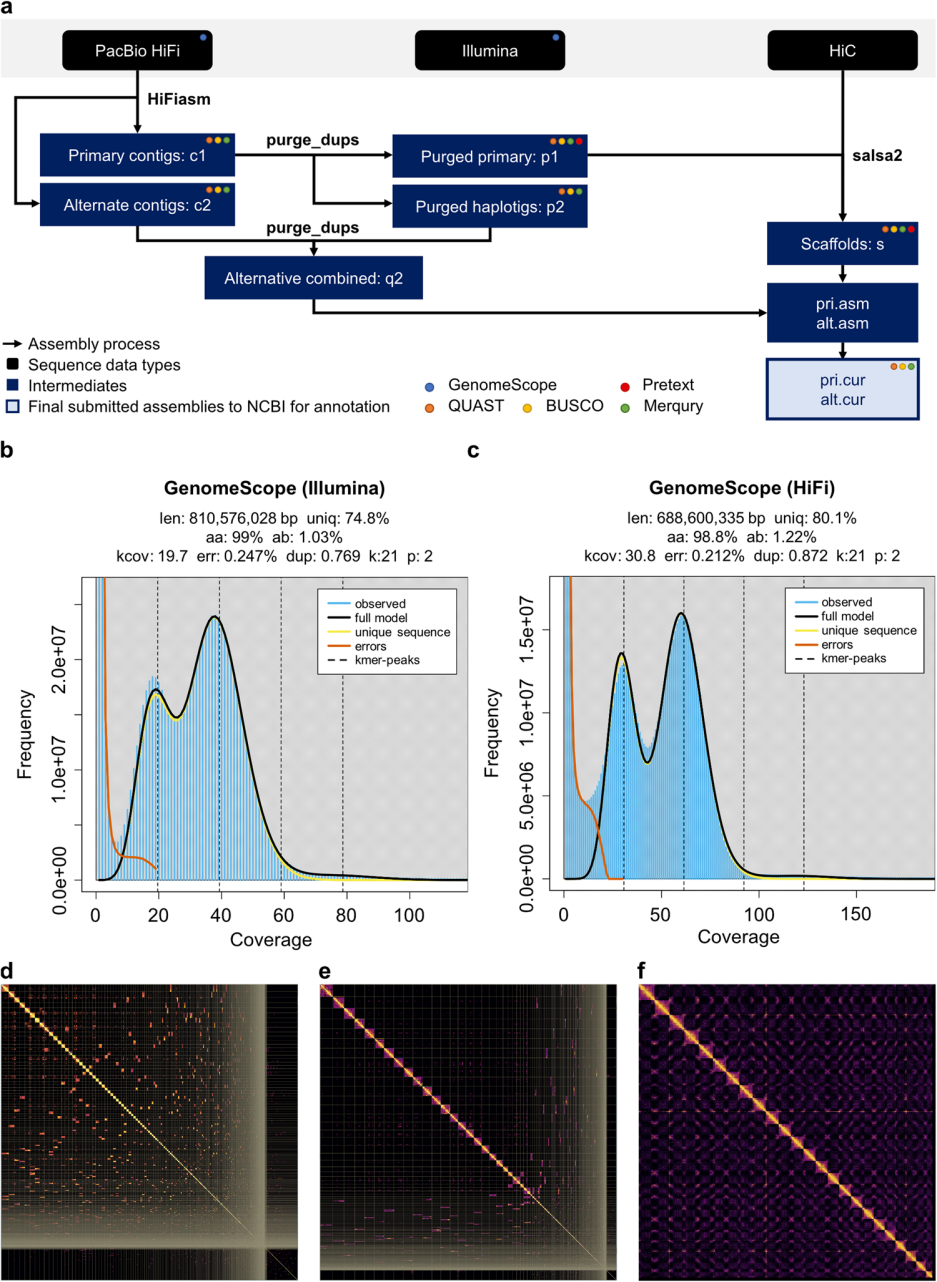
Genome assembly process to build a reference genome of chub mackerel (fScoJap1). (**a**) VGP standard assembly pipeline v.2.0. with PacBio HiFi and Arima Hi-C data. Transformed linear GenomeScope profile plots of fScoJap1 genome generated with Illumina short reads (**b**) and PacBio HiFi reads (**c**). Pretext contact Hi-C maps of the duplication-removed contigs of fScoJap1 named as ‘p’ (**d**), the scaffolds of fScoJap1 linked by Hi-C named as ‘s’ (**e**) and the final curated assembly of fScoJap1, ordered by chromosome numbers, named as ‘pri.cur’ (**f**).

**Fig. 3:**
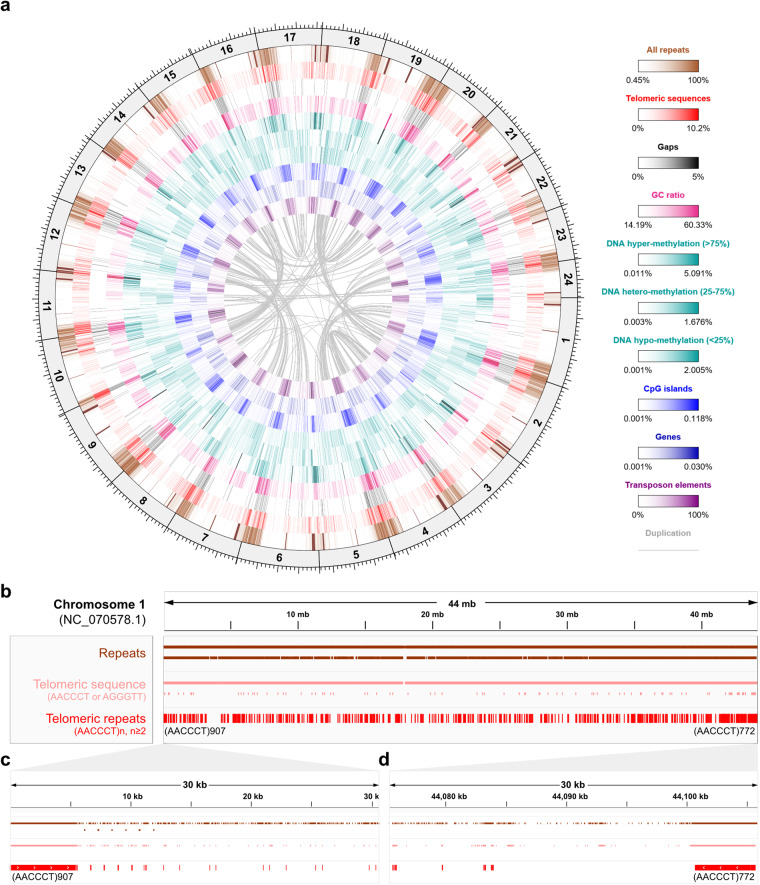
Chromosome-level scaffolds in fScoJap1 genome assembly. (**a**) Circos plot of 24 chromosomes. From the outermost track, each track represents: chromosome lengths, all repeats, telomeric repeats, gaps, GC content, likely methylated CpG sites, moderately likely methylated CpG sites, unlikely methylated CpG sites, CpG islands, genes, DNA transposon elements and synteny links. The coordinate of the circos plot is indicated by the ticks on the chromosomal (the outermost) track. Each minor tick on the outer side of the chromosome represents 2 Mbp and each major tick represents 10 Mbp. All tracks are quantile scaled. For each track, the intensity of color represents the percentage of the bases occupied by the feature in every 100,000 bp window of the corresponding region of the genome. (**b**) repeats and telomeric repeats in chromosome 1. c. telomeric sequences at 5′ (**c**) and 3′ (**d**) ends in chromosome 1.

**Fig. 4:**
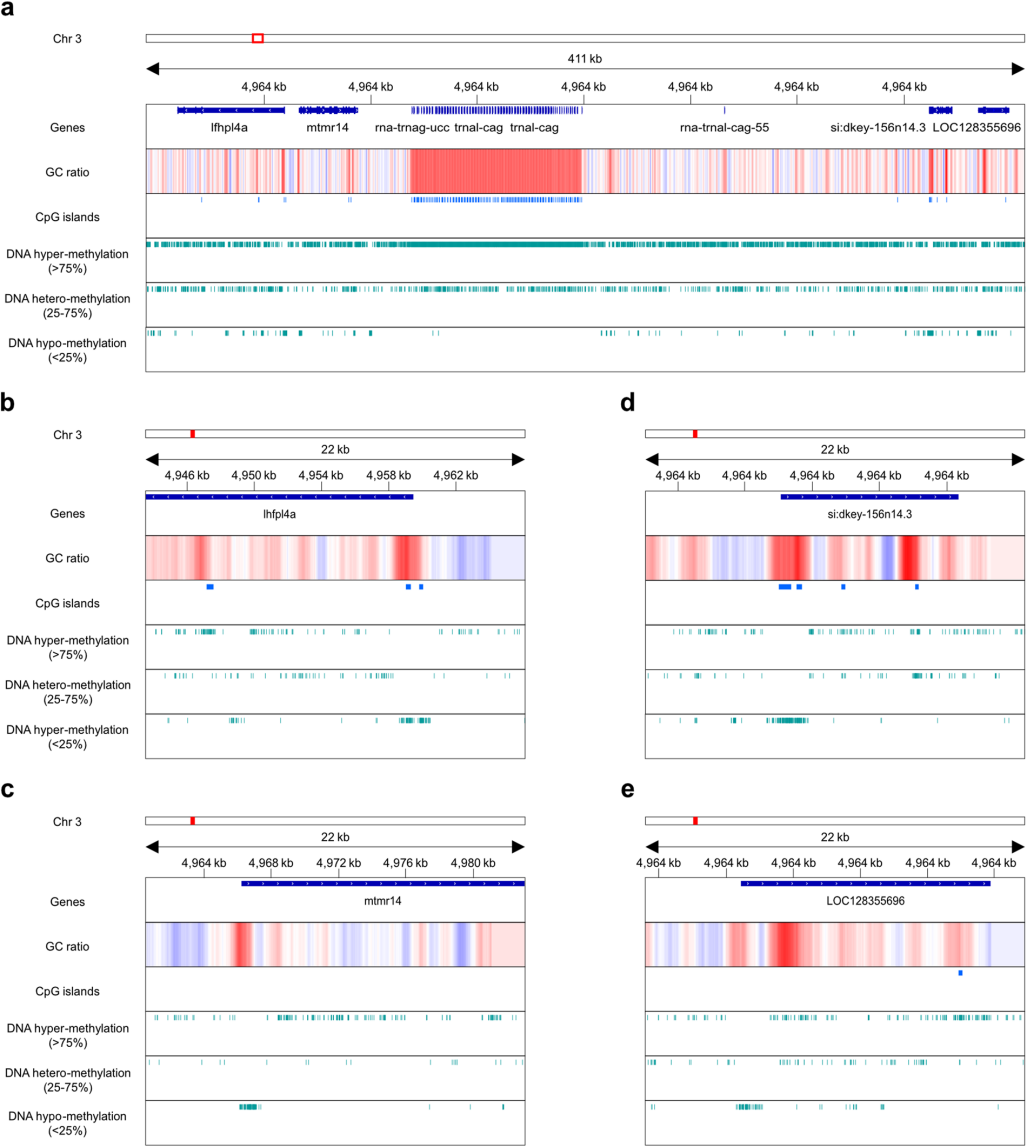
DNA hyper/hypo-methylation profiles on a tRNA cluster and its neighbor genes. Local view of loci in chromosome 3. (**a**) chr3:4,894,502–5,306,197 of fScoJap1 genome and promoters of vicinal genes on 5′ direction (**b**) chr3:4,943,527–4,966,068, (**c**) chr3:4,960,500–4,983,041) and 3′ direction (**d**) chr3:5,254,050–5,276,591, (**e**) chr3:5,279,191–5,301,732). In descending order, each track pertains to genes, CpG islands and CpG sites with three different classes of 5mC modification probabilities (>75%, 25–75% and <25%, respectively). Direction of arrowheads on gene blocks indicates coding strand orientation of gene.

**Fig. 5:**
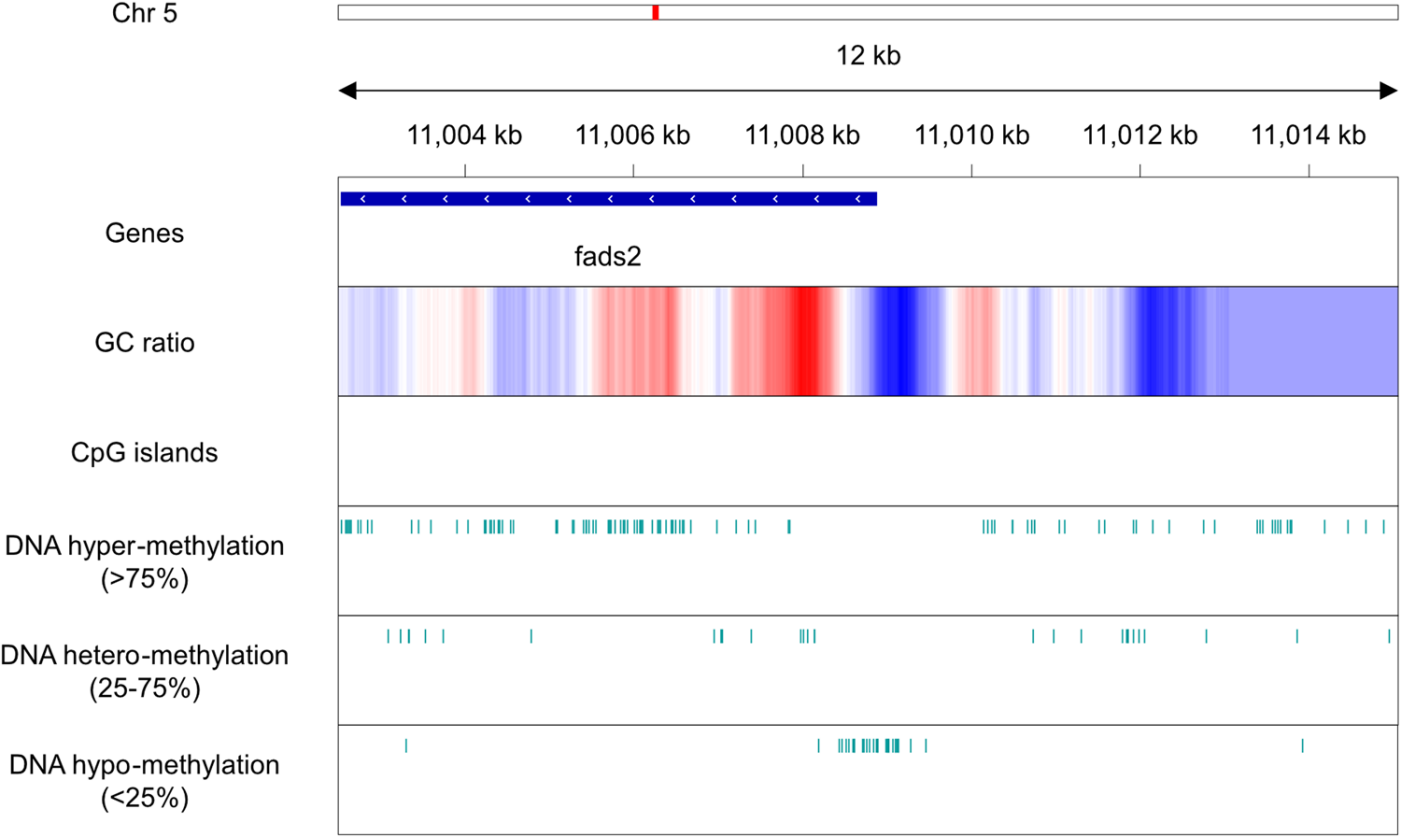
DNA hypo-methylations on the promoter of *Fads2* gene. Local view of 12 kb region on chromosome 5:11,002,496–11,015,040 of fScoJap1 genome containing the promoter region of *Fads2* gene. In descending order, each track pertains to genes, CpG islands, and CpG sites with three different classes of 5mC modification probabilities (>75%, 25–75% and <25%, respectively).

Here, we constructed a chromosome-level genome assembly of a male chub mackerel individual (fScoJap1) collected from the South Sea of South Korea (Fig. 1c). We extracted genomic DNA from five different tissues and performed sequencing using PacBio long high-fidelity (HiFi), Illumina and Arima Hi-C technologies, following the Vertebrate Genomes Project (VGP) assembly standard pipeline v2.0^13,14^ (Fig. 2a). The estimated genome size using GenomeScope^15^ on Illumina genomic reads was 810 Mb (Fig. 2b), while on HiFi reads was shorter (628 Mb) (Fig. 2c). The underestimation of genome size with HiFi reads is consistent with patterns seen in other recent high-quality genome assemblies^16–22^ (Supplementary table S2), most prominent in teleost fishes (*Actinopterygii*). The recent study on the HiFi assembly of the closest species to chub mackerel, Atlantic chub mackerel, only made a genome size estimation using Illumina reads^23^. The Hi-C mapping allowed reflection of 3D structural distances within each chromosome (Fig. 2d,e). We assembled genome sequences totalling 828.68 Mb in length, which is comparable to the 814.07 Mb assembly of its closest relative, Atlantic chub mackerel^23^. The assembly yielded 24 distinct chromosomal scaffolds (Fig. 2d, Table 1) mostly supported by telomeres at their 5′ and 3′ ends, except for chromosome 10 (Fig. 3, Table 2). We annotated a total of 31,656 genes, including 30,506 protein-coding genes (Table 3) and observed suppression of DNA transposon elements within duplicated genes (Fig. 3a). By examining the 5-methylcytosine (5mC) profile in gene promoter regions using HiFi read data, we gained insight into the open/close chromatin structures associated with a tRNA cluster (Fig. 4) and Omega-3 production genes (Fig. 5). Overall, the chub mackerel genome assembled in this study represents a valuable genetic resource with implications for various fields, including biology, industry, and conservation.

**Table 1:** Summary statistics of fScoJap1 assembly.

	Contig	Scaffold
Number	1,932	360
N50 (bp)	4,898,551	34,636,535
L50	46	11
Total length (bp)	828,681,152	
Total ungapped length (bp)	828,034,052	
Chromosomal scaffolds	24	
Total length of all chromosomal scaffolds (bp) (percentage in genome)	819,043,197 (98.84%)	

More details in supplementary table S3.

**Table 2:** Telomeres at 5′ and 3′ ends of chromosomes in fScoJap1 assembly.

Chromosome	5′ end (30kbp)			3′ end (30kbp)		
	Repeats (bp)	Telomeric ‘AACCCT’ or ‘AGGGTT’ (bp)	Repeats with telomeric seq. (bp)	Repeats (bp)	Telomeric ‘AACCCT’ or ‘AGGGTT’ (bp)	Repeats with telomeric seq. (bp)
1	17,337	6,324	6,217	12,459	5,928	5,748
2	20,815	240	112	17,030	5,322	5,077
3	11,366	1,422	1,340	4,470	96	—
4	15,879	1,806	1,746	18,599	102	36
5	9,535	690	567	18,475	4,458	4,295
6	22,903	7,494	7,426	13,767	1,146	1,066
7	15,560	5,262	5,112	16,637	7,380	3,768
8	8,047	540	350	9,576	1,854	1,558
9	12,498	4,740	4,696	8,619	534	474
10	13,415	1,242	901	—	6	—
11	12,908	132	66	13,302	1,422	1,316
12	7,825	156	120	11,607	5,262	5,184
13	21,391	5,832	5,741	23,179	126	60
14	20,847	1,692	1,554	11,757	822	616
15	14,940	4,152	4,081	7,036	438	366
16	9,490	684	642	14,058	3,702	3,538
17	18,450	4,020	4,017	22,687	4,392	4,344
18	21,313	24	18	11,622	894	770
19	19,369	3,714	3,613	24,277	4,014	3,845
20	16,972	1,158	640	14,077	114	33
21	16,707	2,742	2,586	13,604	4,380	4,310
22	7,486	6,246	6,219	17,591	5,724	5,521
23	7,659	672	543	9,352	1,224	1,103
24	16,915	4,674	4,548	9,894	1,206	1,083

**Table 3:** Gene annotation of fScoJap1 assembly.

Feature	Number	Mean length (bp)	Minimum length (bp)	Maximum length (bp)
Genes	31,656	13,356	57	592,634
Transcripts	38,903	2,086	57	98,580
mRNA	30,506	2,601	102	98,580
misc_RNA	240	2,602	97	14,185
tRNA	4,513	74	69	99
lncRNA	944	443	68	5,598
snoRNA	267	124	57	347
snRNA	905	150	57	192
rRNA	1,521	163	118	4,030
CDS	30,506	1,911	102	98,187
Exons	258,465	228	1	17,325
Introns	233,067	1,682	30	543,104
Mean transcripts per gene	1.27			
Mean exons per transcript	10.45

## Methods

### Sample collection, library construction, and sequencing

Brain, gill, muscle, liver and gonad tissues of a male chub mackerel caught in juvenile stage and farmed in Se-Bo Su-San near Dara National Park, Gyeongsangnam-do, South Korea (34°46′15.8″ N, 128°23′54.0″ E) (Fig. 1c) were collected on July, 2019. Samples were stored at −80 °C until genomic DNA was extracted using Circulomics Nanobind Tissue Big DNA Kit from brain and muscle tissues for PacBio HiFi and Arima Hi-C sequencing, respectively. We anaesthetized the animal with ethanol and sacrificed with guillotine to minimize pain, followed by tissue dissections; all protocols followed the guideline for animal care of Pukyong National University. Quantity and quality of DNA was determined by Qubit 3 Fluorometer and Agilent Fragment Analyzer. Two PacBio HiFi libraries with insert size of 16,000 bp were generated with 7.5 μg of genomic DNA using SMRTbell® express template prep kit 2.0. The library was sequenced on a PacBio Sequel II system and 44 Gb of HiFi (QV ≥ 20) data was generated with 49 × coverage and an average read length of 14,000 bp^24^. Additionally, 80.68 Gb of Hi-C data with 89.64 × coverage from the same sample was generated with Arima Hi-C v2.1^24^ (Table 4).

**Table 4:** Raw sequencing data of fScoJap1.

	PacBio HiFi (Q ≥ 20)	Arima Hi-C	Illumina
Bases (Gbp)	44.3	80.7	39.0
Coverage (X)	49.2	89.6	43.3
Bytes (GiB)	18.2	53.3	18.6
Link	https://genomeark.s3.amazonaws.com/index.html?prefix=species/Scomber_japonicus/fScoJap1/genomic_data/pacbio_hifi/	https://genomeark.s3.amazonaws.com/index.html?prefix=species/Scomber_japonicus/fScoJap1/genomic_data/arima/	https://genomeark.s3.amazonaws.com/index.html?prefix=species/Scomber_japonicus/fScoJap1/genomic_data/illumina/

### Geographical distribution map

Integrated information of every recorded occurrence of chub mackerel was retrieved from Ocean Biodiversity Information System (OBIS) database^25^. Citations for subsets of every dataset are provided in Supplementary table S1. The geographic distribution map (Fig. 1b,c) was visualized using rnaturalearth package^26^ for R^27^ by plotting coordinate information of OBIS data for mackerel occurrences on the world map.

### Genome assembly

The fScoJap1 genome was assembled through VGP standard pipeline v2.0 (https://training.galaxyproject.org/training-material/topics/assembly/tutorials/vgp_genome_assembly/tutorial.html)^13,14^ (Fig. 2a). Bionano optical mapping was excluded because it did not produce sufficient quality long-molecule maps, which occurs for some species. The genome size was estimated to be 810,576,028 bp and 688,600,335 bp by GenomeScope^15^ with *k* = 21 using Illumina and HiFi unassembled reads^24^, respectively (Fig. 2b,c). The tendency for genome size to be substantially underestimated when predicted by HiFi reads is prevalent in other species of various lineages^16,17^, with the biggest differences seen in fish^18–22^ (Supplementary table S2). Such discrepancies are likely due to genomic regions that HiFi provides less coverage compared to Illumina^28^. Nonetheless, those regions are constructed with high accuracy in the final genome assembly, and thus the final genome size (Table 1) is larger than that predicted using HiFi reads (Fig. 2c) and closer to that predicted using Illumina reads (Fig. 2b).

First, primary (c1) and alternate (c2) contigs were generated by HiFiasm^29,30^ with HiFi reads^24^. QUAST^31^ analysis indicated that c1 comprised a total of 4,037 contigs (N50 = 4,041,932 bp). BUSCO^32^ analysis indicated that 3,587 of 3,640 conserved single-copy genes in Actinopterygii (v5.4.7) vertebrates were present in the c1 assembly, of which 468 were single-copies, 3,095 were duplicated and 24 were fragmented. QV and completeness evaluated using Merqury^33^ were 58.0052 and 98.5075%, respectively for c1; 59.0171 and 10.7859%, respectively for c2; and 58.0576 and 99.7446%, respectively for c1 + c2 (Supplementary table S3).

Second, false haplotype duplicate sequences were removed from the primary contigs to generate purged primary contigs and haplotigs (c1 → p1, p2) using purge_dups v1.2.5^34^; the purged haplotigs were added to the alternate assembly (c2, p2 → q2). QUAST analysis after purging indicated that p1 and p2 each comprised totals of 1,922 (N50 = 5,024,282 bp) and 2,156 (N50 = 2,259,549 bp) contigs, respectively. BUSCO analysis after purging indicated that 3,593 of 3,640 conserved Actinopterygii genes were present in the p1 assembly, of which 3,494 were single-copies, 64 were duplicated and 35 were fragmented (Supplementary table S4). QV and completeness evaluated using Merqury were 57.7529 and 85.3721%, respectively for p1; 58.6418 and 83.8403%, respectively for p2; and 58.1599 and 99.557%, respectively for p1 + p2 (Supplementary table S3).

Third, the remaining primary contigs were scaffolded (p1 → s) using Hi-C data with salsa v2.3^35,36^ (Fig. 2d,e). Only the primary assembly (p1) was scaffolded, as the alternate (p2) contains just the alternate haplotype pieces of contigs that are not as complete as the primary. QUAST analysis after Hi-C scaffolding indicated that s comprised a total of 762 contigs (N50 = 22,224,178 bp). QV and completeness evaluated using Merqury were 23.2014 and 99.8512%, respectively for s (Supplementary table S3).

Last, the draft assembly was decontaminated and manually curated using gEVAL v2.2.0^37^ (Fig. 2f). After 69 breaks, 463 joins and removal of 7 erroneously remaining duplicated contigs, the scaffold N50 was increased by 56% to 34.6 Mb and the scaffold count reduced by 53% to 360. Of the manually curated assembly, 98.9% could be assigned to 24 identified chromosomes, which were named according to synteny with the closely related Thunnus maccoyii (Southern bluefin tuna) assembly GCF_910596095.1. After manual curation, the curated assembly was 828,697,720 bp, containing 361 scaffolds with a scaffold N50 of 34,636,535 bp (Supplementary table S3). The manually curated assembly was uploaded on GenBank under accession GCA_027409825.1^38^, where the NCBI team removed some microbial contaminating contigs. The further decontaminated assembly was 828,681,152 bp, containing 1,932 contigs with contig N50 of 4,898,551 bp and 360 scaffolds with scaffold N50 of 34,636,535 bp (Table 1, Supplementary table S3). NCBI annotated this assembly under accession GCF_027409825.1^39^. All downstream analyses were carried out on the final assembly.

### Telomeric repeats

Number of telomeric repeats for every 10,000 bp windows of the genome were identified with tidk v0.2.1 (https://github.com/tolkit/telomeric-identifier) by searching for forward and reverse matches with the telomeric repeat sequence for the *Scombriformes* clade (‘AACCCT’) obtained from the telomeric repeat database (http://telomerase.asu.edu/sequences_telomere.html). Soft-masked repeats and telomeric sequences located on telomeric regions (30 kb ends of chromosomes) of every chromosome were counted by an in-house Python script (https://github.com/chulbioinfo/fScoJap1)^40^.

To evaluate if chromosomes were properly assembled and partitioned, we investigated telomeric repeats at the ends of each chromosome. 437,667 occurrences of telomeric repeat sequence for the *Scombriformes* clade ‘AACCCT’ or its complementary ‘AGGGTT’ were identified throughout the genome with tidk. With an exception of the 3′ telomere of chromosome 10, all chromosomal telomeres of fScoJap1 assembly contained the telomeric repeat sequences (Fig. 3a, Table 2), suggesting that chromosomes were properly assembled end to end. For example, chromosome 1 had 907 and 772 copies of (ACCCTT)n telomeric repeats at the 5′ and 3′ ends, respectively (Fig. 3b–d).

### Repeat annotation

All repetitive regions of the fScoJap1 genome were located, soft-masked and incorporated in the assembly with WindowMasker^41^. Specific repetitive elements and their numbers were identified with RepeatMasker v4.1.5^42^ using Dfam v3.7^43^ library for zebrafish (*Danio rerio*).

Overall, 261,419,747 bp of sequences composing 31.55% of the assembly were masked as repeats by WindowMasker (Fig. 3a). Repetitive elements classified as specific repeat classes and families identified by RepeatMasker totaled 111,477,307 bp (Table 5), including 144,914 DNA transposons, totalling 18,619,431 bp. There was an overall tendency for repetitive elements to be concentrated at the telomeric regions of chromosomes (Fig. 3a).

**Table 5:** Repetitive elements of fScoJap1 assembly.

	# of elements	Length (bp)	Percentage in genome
SINE	4,853	547,707	0.07%
LINE	46,756	1,591,223	1.93%
LTR	25,858	7,160,831	0.86%
DNA transposons	144,914	18,619,431	2.25%
Unclassified	501	237,532	0.03%
Small RNA	10,051	1,129,995	0.14%
Satellites	2,856	423,675	0.05%
Simple repeats	559,439	50,350,567	6.08%
Low complexity	84,541	14,512,782	1.75%

### Gene annotation

The assembled fScopJap1 genome was annotated through NCBI Eukaryotic Genome Annotation Pipeline v10.1^44^. For gene prediction, experimental evidences retrieved from Entrez Nucleotide, Entrez Protein and SRA of NCBI were aligned to the fScoJap1 genome. 52 GenBank transcripts and 304 EST sequence data from dbEST of chub mackerel were aligned using Splign^45^. RNA-Seq reads from 11 chub mackerel liver samples (NCBI Accession: SAMN08995495, SAMN08995496, SAMN08995497, SAMN08995498, SAMN08995499, SAMN08995500, SAMN08995501, SAMN08995502, SAMN08995503, SAMN08995504, SAMN10118436), one Atlantic chub mackerel liver sample (NCBI Accession: SAMN08159728), one Atlantic mackerel (*Scomber scombrus*) liver sample (NCBI Accession: SAMN12342693) and one Atlantic mackerel white muscle sample (NCBI Accession: SAMN04992872) were aligned using STAR^46^. RefSeq proteins of siamese fighting fish (*Betta splendens*), ray-finned fish (*Actinopterygii*), zebrafish, northern pike (*Esox lucius*), southern platyfish (*Xiphophorus maculatus*) and human (*Homo sapiens*) and GenBank proteins of ray-finned fish and human were aligned using ProSplign^47^. The annotation was uploaded on NCBI RefSeq with annotation ID “GCF_027409825.1-RS_2023_01.”

### Duplication

Duplicated genes were identified using a wrapper for MCScanX^48^ provided in TBtools-II v1.113^49^ by searching for BLASTP matches within the fScoJap1 genome with the number of BLASTP hits for a gene restricted to five and an E-value cutoff set to 10^−10^. Only coding sequences (CDSs) with start and stop codons which totalled to 23,774 were analyzed and further classified according to a classification procedure by Wang *et al*.^48^: WGD/segmental if it is an anchor gene in a collinear duplication; tandem duplicates if the corresponding duplicate is the gene adjacent on the chromosome; proximal if the duplicate is less than 20 genes apart; and dispersed for every other duplicated genes (Table 6).

**Table 6:** Gene duplications in fScoJap1 assembly.

Type of duplications	Genes
Singleton	3,780
Dispersed	13,158
Proximal	1,092
Tandem	2,873
WGD/segmental	2,871
Total	23,774

A total of 19,994 genes contain various duplications classified into 13,158 dispersed, 1,092 proximal, 2,873 tandem and 2,871 WGD/segmental duplications, respectively (Table 6). Visual inspection of the circus plot suggested an overall tendency for genic duplications to be less in regions of the genome where transposons were located (Fig. 3a). To quantify this, we calculated the total length of transposons in duplicated genic regions of the genome compared to other regions. Whole genic regions had lower proportion overlapped with transposon elements (2.03%) than did whole intergenic regions (2.56%). Within the genic regions, the percentage of duplicated genic regions covered by transposon elements (1.30%) were almost twice as less than the percentage of singleton genic regions covered by transposons (2.37%; Table 7), suggesting a disposition of transposons to overlap less with duplicated genes. This finding is intriguing, as it is counterintuitive to the fact that transposons are in part responsible for forming new gene duplications^50^.

**Table 7:** Regions overlapped by transposon elements for duplicated genes with respect to other genes.

	Whole genic	Whole intergenic	Singleton genic	Duplicated genic
Total length (bp)	411,502,902	417,178,250	41,523,569	53,214,346
Number of TE overlaps	61,923	73,050	6,999	5,929
Length of TE overlaps (bp)	8,367,540	10,685,012	982,812	690,859
Percentage covered by TE	2.03%	2.56%	2.37%	1.30%

### GC content and DNA methylation

Methylation profiles were identified by kinetic signatures imprinted on HiFi reads which specify positions of CpG sites and probabilities of 5mC modifications. The 5mC modification information of HiFi reads were read by primrose v1.3.0^51^ which generated an identical set of HiFi reads with the information tagged as BAM tags. The tagged reads were aligned to the chub mackerel assembly, sorted and indexed by pbmm2 v1.10.0 (https://github.com/PacificBiosciences/pbmm2). Complete list of CpG sites and their 5mC modification probabilities based on the aligned tagged reads were generated by pb-CpG-tools v1.1.0 (https://github.com/PacificBiosciences/pb-CpG-tools/), which calculated discretized modification probabilities based on the estimated ratio of reads mapped to the corresponding CpG site tagged as modified to those tagged as not modified. CpG islands were identified by ‘newcpgreport’ function of EMBOSS: 6.5.7.0 (http://emboss.bioinformatics.nl/cgi-bin/emboss/newcpgreport).

Genes are known to have differential methylation of CpG islands on promoters which affect transcription initiation in many genes^52^. All CpG sites were located and further classified as hyper- (>75%), hetero- (25%~75%) or hypo-methylated (<25%) discretized from 5-methylcytosine (5mC) modification probability. In total, 10,636,128 CpG sites were identified, of which 7,271,538 were likely, 2,108,856 were moderately likely, and 1,255,734 were unlikely methylated (Fig. 3a). A total of 35,728 CpG islands were found throughout the genome which summed to 10,839,030 bp in length (Fig. 3a).

A substantial number of CpG sites were found located on genes or supposable promoter regions of genes (≤1,000 bp upstream of transcription initiation site; Fig. 3a). For example, we found 118 CpG islands each covering one of 158 tRNA genes clustered in an approximately 80,000 bp long region between loci 5,019,165 and 5,098,985 bp on chromosome 3 (3:5,019,165–5,098,985) of the fScoJap1 genome (Fig. 4a). Such case is accordant with an observed tendency for human tRNA genes to have relatively short CpG islands located on them that cover all of the transcription units^53^. Whereas the CpG islands on the tRNA cluster 3:5,019,165–5,098,985 were heavily methylated, apparent by overall skew of CpG sites in the region towards being likely methylated (Fig. 4a), the CpG islands on promoter regions of several nearby genes of the chromosome were relatively unmethylated (Fig. 4b,d). For some genes, although the promoter region lacked a CpG island, the CpG sites at those regions were unmethylated (Fig. 4c,e). Such cases imply non-repression of expressions of those genes^54^.

The DNA hypo-methylation on promoters imply possibilities for new biological insights. For example, the *Fads2* gene (located on 5:11,002,529–11,008,894 in fScoJap1 genome) is expected to be highly expressed in the chub mackerel because it is known to be associated with synthesis of docosahexaenoic acid (DHA), a type of omega-3, a polyunsaturated fatty acid^55^ and a highly-valued nutritional component of chub mackerel. *Fads2* genes code for desaturase enzymes to synthesize long-chain polyunsaturated fatty acids including DHA by introducing double bonds to endogenous fatty acids, causing them to become polyunsaturated^56^. Accordingly, we found the promoter region of *Fads2* gene to be relatively non-methylated (Fig. 5).

## Data Records

The genomic PacBio sequencing and Hi-C data were deposited in NCBI under accession number SRP470260^24^ and GenomeArk (https://www.genomeark.org/vgp-curated-assembly/Scomber_japonicus.html). The assembled genome and genome annotation information was deposited in NCBI GenBank under accession number GCA_027409825.1^38^ and NCBI RefSeq under accession number GCF_027409825.1^39^ (https://www.ncbi.nlm.nih.gov/assembly/GCF_027409825.1).

## Technical Validation

After each step of the assembly procedure, quality control metrics were computed by QUAST v5.0.2, BUSCO v5.4.7 and Merqury v1.3 (Supplementary table S3). BUSCO was run on “genome mode” with Actinopterygii_odb10 lineage dataset (https://busco.ezlab.org/list_of_lineages.html). Merqury analysis was carried out using database (meryldb) generated by Meryl v1.3^33^.

QUAST and BUSCO was run on intermediate assemblies and the final curated fScoJap1 primary assembly for validation of the genome quality. QUAST analysis results indicated that N50 of the final assembly was 34,636,535 bp, concordant with our scaffold N50 (Supplementary table S3). BUSCO analysis results indicated that 3,598 of 3,640 conserved single-copy genes in vertebrata were present in the final assembly, of which 3,537 were single-copies, 34 were duplicated, and 27 were fragmented (Supplementary table S3).

Genes of fScoJap1 assembly were predicted via model-based and ab initio procedures with Gnomon^57^ using an HMM-based algorithm to build annotation “GCF_027409825.1-RS_2023_01.” The final gene set contained 31,656 genes with a mean length of 13,356 bp. Mean lengths of coding sequences (CDSs), exons and introns were 1,911, 228 and 1,682, respectively. There was a total of 258,465 exons in the genome and the mean number of exons per gene was 13.2715 (Table 3). BUSCO was run on “protein” mode using actinopterygii_odb10 lineage dataset (https://busco.ezlab.org/list_of_lineages.html) to assess the completeness of the prediction of gene annotation “GCF_027409825.1-RS_2023_01.” Results of BUSCO analysis yielded a value of 99.1% (complete = 98.4%, single-copy = 97.3%, duplicated = 1.1%, fragmented = 0.7%, missing = 0.9%, genes = 3,640) (Table 8).

**Table 8:** BUSCO scores of fScoJap1 assembly.

	Complete		Fragmented	Missing
	Single-copy	Duplicated		
Percentage	98.4%		0.7%	0.9%
	97.3%	1.1%		
Total groups searched	3,640			

### Supplementary information

Supplementary Table S1

Supplementary Table S2

Supplementary Table S3

Supplementary Table S4

## Data Availability

The software versions, settings and parameters used are described below: 1. GenomeScope v2.0; p = 2, k = 21 2. HiFiasm v0.15.4-r343; ran on Galaxy with default parameters, with the exception of purging level = 0 (none). 3. QUAST v5.0.2; python quast.py [Assembly file name] 4. BUSCO v5.4.7; busco -i [Assembly file name] -l vertebrata_odb10 -m genome 5. Meryl v1.3; (meryldb generation) Meryl was run on all four raw read files separately to generate a meryl database for that sequencing run, and then the four meryl databases were merged using the “union-sum” function, to make a meryl database for all the reads. The *k* value was 21 for all runs. 6. Merqury v1.3; ran on Galaxy with following parameters; Evaluation mode: Default mode, k-mer counts database: fScoJap1.meryldb.meryldb, Number of assemblies: One assembly (“Two assemblies” for running on c1 & c2 simultaneously), Genome assembly: [Assembly file name] 7. purge_dups v1.2.5; ran on Galaxy using workflow “VGP purge assembly with purge_dups pipeline”; Hifiasm Primary assembly: fScoJap1_c1.fasta, Hifiasm alternate assembly: [fScoJap1_c2.fasta] 8. salsa v2.3; ran on Galaxy with parameters; Initial assembly file: p1.fastq, Bed alignment: Aligned bed format files of Hi-C data (fScoJap1_S_2476_8_R1_001.fasta, fScoJap1_S_2476_8_R2_001.fasta) 9. gEVAL v2.2.0; 10. RepeatMasker v4.1.5; ran with following parameters; Repeat library source: Dfam 3.7, Species: zebra fish; Search engine: RMBlast v2.14.0 + ; Sensitive search option. 11. tidk v0.2.1; tidk find -c Scombriformes -f [GCF_027409825.1_fScoJap1.pri_genomic.fna] -w 10000 12. primrose v1.3.0; primrose [fScoJap1_HiFi.bam fScoJap1_5mC-HiFi.bam] 13. pbmm2 v1.10.0; pbmm2 index [GCF_027409825.1_fScoJap1.pri_genomic.fna] fScoJap1_5mC-HiFi.bam fScoJap_5mC-HiFi.mmi; pbmm2 align [fScoJap1_5mC-HiFi.mmi fScoJap_5mC-HiFi.bam] [fScoJap1_5mC-HiFi_aligned_sorted.bam]–sort 14. pb-CpG-tools v1.1.0; python aligned_bam_to_cpg_scores.py -b [fScoJap_5mC_HiFi_aligned_sorted.bam] -f [GCF_027409825.1_fScoJap1.pri_genomic.fna] -o cpg_regions -p model -d /pileup_calling_model/ 15. EMBOSS v6.5.7.0; newcpgreport -window 100 -shift 1 -minlen 200 -minoe 0.6 -minpc 50. [GCF_027409825.1_fScoJap1.pri_genomic.fna] 16. TBtools-II v1.113; ran in GUI through Graphics > Comparative Genomics > One Step MCScanX option with following parameters; Input Genome Sequence File (.fa) of Species One: GCF_027409825.1_fScoJap1.pri_genomic.fna, Input Gene Structure Annotation File (.gff/.gtf3) of Species One: GCF_027409825.1_fScoJap1.pri_genomic.gff, Input Genome Sequence File (.fa) of Species Two: GCF_027409825.1_fScoJap1.pri_genomic.fna, Input Gene Structure Annotation File (.gff/.gtf3) of Species Two: GCF_027409825.1_fScoJap1.pri_genomic.gff, CPU for BlastP: 2, E-value: 1e-10, Num of BlastHits: 5 17. BUSCO v4.1.4; ran on RefSeq annotation “GCF_027409825.1-RS_2023_01” with following parameters; Lineage: actinopterygii_odb10, Mode: Protein No custom scripts or code was used in validation of the dataset.
